# The Presence of D-Penicillamine during the In Vitro Capacitation of Stallion Spermatozoa Prolongs Hyperactive-Like Motility and Allows for Sperm Selection by Thermotaxis

**DOI:** 10.3390/ani10091467

**Published:** 2020-08-21

**Authors:** Sara Ruiz-Díaz, Ivan Oseguera-López, David De La Cuesta-Díaz, Belén García-López, Consuelo Serres, Maria José Sanchez-Calabuig, Alfonso Gutiérrez-Adán, Serafin Perez-Cerezales

**Affiliations:** 1 Department of Animal Reproduction, National Institute for Agriculture and Food Research and Technology (INIA), 28040 Madrid, Spain; sruizd@clinicatambre.com (S.R.-D.); davedlcd@gmail.com (D.D.L.C.-D.); belen.garcia@inia.es (B.G.-L.); serafin.perez@inia.es (S.P.-C.); 2 Mistral Fertility Clinics S.L., Clínica Tambre, 28002 Madrid, Spain; 3 Unidad Iztapalapa, Universidad Autónoma Metropolitana, Ciudad de México 09340, Mexico; ivanoslo@yahoo.com.mx; 4 Departamento de Medicina y Cirugía Animal, Facultad de Veterinaria, Universidad Complutense de Madrid (UCM), 28040 Madrid, Spain; cserres@ucm.es (C.S.); msanch26@ucm.es (M.J.S.-C.)

**Keywords:** stallion sperm, capacitation, penicillamine, thermotaxis, selection

## Abstract

**Simple Summary:**

Capacitation of stallion semen in vitro is still a suboptimal procedure. The main objective of this study was the use of thermotaxis as a novel method for sperm selection and determining the most adequate media for maintaining frozen/thawed horse sperm longevity in vitro. Our results show that the most common media (Whitten’s) used in this species is not the best for capacitating the semen in terms of hyperactive-like motility, and tyrosine phosphorylation being synthetic human tubal fluid supplemented with D-penicillamine is the most adequate in preserving these parameters during 180 min of incubation. Therefore, this media (with and without D-penicillamine) was chosen for performing thermotaxis. The selection conditions were a gradient of 3 °C of difference (35–38 °C) for 1 h. The results revealed that the selected fraction showed higher levels of tyrosine phosphorylation in the whole flagellum and lower levels of DNA fragmentation when compared to the unselected fraction (kept at 37 °C) when human tubal fluid with D-penicillamine was used. These results are promising for improving the in vitro embryo production rates in these species by improving the sperm selection methodology.

**Abstract:**

Assisted reproductive technologies (ARTs) in the horse still yield suboptimal results in terms of pregnancy rates. One of the reasons for this is the lack of optimal conditions for the sperm capacitation in vitro. This study assesses the use of synthetic human tubal fluid (HTF) supplemented with D-penicillamine (HTF + PEN) for the in vitro capacitation of frozen/thawed stallion spermatozoa by examining capacitation-related events over 180 min of incubation. Besides these events, we explored the in vitro capacity of the spermatozoa to migrate by thermotaxis and give rise to a population of high-quality spermatozoa. We found that HTF induced higher levels of hyperactive-like motility and protein tyrosine phosphorylation (PTP) compared to the use of a medium commonly used in this species (Whitten’s). Also, HTF + PEN was able to maintain this hyperactive-like motility, otherwise lost in the absence of PEN, for 180 min, and also allowed for sperm selection by thermotaxis in vitro. Remarkably, the selected fraction was enriched in spermatozoa showing PTP along the whole flagellum and lower levels of DNA fragmentation when compared to the unselected fraction (38% ± 11% vs 4.4% ± 1.1% and 4.2% ± 0.4% vs 11% ± 2% respectively, *t*-test *p* < 0.003, *n* = 6). This procedure of in vitro capacitation of frozen/thawed stallion spermatozoa in HTF + PEN followed by in vitro sperm selection by thermotaxis represents a promising sperm preparation strategy for in vitro fertilization and intracytoplasmic sperm injection in this species.

## 1. Introduction

Despite nearly two decades of efforts, in vitro fertilization (IVF) in the horse remains unavailable, and intracytoplasmic sperm injection (ICSI) in this species still yields suboptimal results. One of the main causes of these limitations are suboptimal in vitro conditions for sperm capacitation, preventing successful IVF [1] and low ICSI outcomes [2].

Synthetic media successfully used for the in vitro capacitation of sperm in other mammalian species are also being tested in the stallion. Thus, reports exist of capacitation-related events that occur in equine spermatozoa under different incubation conditions employing the media Biggers–Whitten–Whittingham (BWW), Tyrode’s, Whitten’s, or human tubal fluid [3,4,5,6,7,8]. However, the available data indicate that using these media, spermatozoa are not fully capacitated, with the consequence that in vitro fertilization remains elusive in this species [1]. One of the main events occurring during sperm capacitation in mammals is the phosphorylation of multiple proteins at their tyrosine residues [9]. Specifically, protein tyrosine phosphorylation (PTP) in the sperm flagellum has been related to the hyperactive motility of sperm, and both these factors are considered hallmarks of mammalian sperm capacitation [10,11]. However, while PTP in the equine spermatozoon flagellum is elevated under different capacitating conditions in vitro, so far all attempts to establish its relationship with hyperactive motility have had limited success [12]. Romero-Aguirregomezcorta [13] showed that the type of hyperactivated motion induced in vitro by species-specific hyperactivation agonists significantly differed in stallion in relation to human or ram spermatozoa, and led to the absence of a rheotactic response in stallion sperm. As an explanation, the authors suggested a different role of sperm hyperactivation in the horse. However, we propose here that it could be the suboptimal in vitro conditions for stallion spermatozoa that prevent a true or complete hyperactivation response.

The proportions of mammalian spermatozoa that acquire a capacitated status at a given time point can be low (roughly around 10%) [14,15,16]. Further, in humans, this capacitated state of spermatozoa is transient (>50 min to <4 h) and occurs only once in a sperm’s lifespan [14]. Harrison [17] described capacitation as a series of positive destabilizing events that eventually lead to sperm death. Later on, Aitken et al. [18] related the physiological production of reactive oxygen species (ROS) to capacitation, followed by apoptosis and sperm senescence. The concept of sperm death as a consequence of capacitation implies that too-high or too-rapid induction of capacitation can shorten the lifespan to the extent that fertilization is prevented [19]. Aitken et al. [20] suggested that the reduced lifespan of spermatozoa incubated in vitro is the outcome of an excessive production of free radicals that eventually provokes lipid peroxidation and the generation of electrophilic cytotoxic aldehydes. To counteract this effect, these authors showed that the addition of penicillamine (PEN), a molecule that neutralizes these aldehydes and slows down their production, improved the maintenance of motility of fresh equine spermatozoa using a non-capacitating medium. In earlier work, Pavlok [21] established that the addition of PEN to the capacitating medium significantly prolonged the lifespan of frozen/thawed bovine spermatozoa, maintaining their fertilization ability for at least 8 h. Accordingly, we hypothesized that capacitated stallion spermatozoa incubated under in vitro conditions for capacitation acquire PTP in their flagella, rapidly losing their viability. This means that physiological hyperactivation is prevented or defective, at least for a short time, and as a consequence, fertilizing ability is lost. This effect could be even more pronounced in some horses because of the initial low quality of their semen [2]. To test this hypothesis, herein we examined the effect of supplementing the capacitating medium with PEN to prolong the lifespan of capacitated equine spermatozoa.

Recently, we proposed that capacitated spermatozoa could be selected from the whole pool of spermatozoa by an in vitro thermotaxis assay [22]. Sperm thermotaxis, defined as the ability of spermatozoa to navigate within a temperature gradient towards the warmer temperature, seems to be exclusive to capacitated spermatozoa [23,24]. In recent work, we found that the DNA of human and mouse spermatozoa selected by thermotaxis in vitro is of very high integrity when compared to the DNA of unselected spermatozoa [22]. Further, the use of these selected spermatozoa significantly increased successful ICSI outcomes in mice. For selection by thermotaxis, capacitated spermatozoa need to preserve their motility during migration within the temperature gradient. This study aimed to evaluate if PEN could prolong the lifespan of frozen/thawed sperm in different capacitating media, thus allowing spermatozoa migration by thermotaxis. Also, this selection could serve to obtain a sperm fraction of high genetic quality for its use in both IVF and ICSI.

The objectives of this study were: (i) to assess the effect of penicillamine supplementation on capacitation-related events during incubation under capacitating conditions in frozen/thawed stallion spermatozoa, (ii) to examine the capacity of these spermatozoa to migrate by thermotaxis, and (iii) to determine DNA integrity and tyrosine phosphorylation in the spermatozoa selected by thermotaxis.

## 2. Materials and Methods

### 2.1. Reagents

All reagents were purchased from Sigma–Aldrich (Saint Louis, MO, USA) unless specified otherwise.

### 2.2. Experimental Design

In an initial experiment, we examined the effect of incubating frozen/thawed stallion spermatozoa processed by density gradient centrifugation (DGC) in Whitten’s medium (WHI) and synthetic human tubal fluid (HTF), two media commonly used for the capacitation of mammalian spermatozoa. Because in our preliminary experiments HTF induced more signs of capacitation (confirmed in the experiments shown here), we supplemented it with 750 µM of penicillamine (HTF + PEN), as this concentration has been shown to prolong sperm motility in the horse [20]. Over an incubation period of 180 min, we evaluated the sperm integrity and capacitation, analyzing the plasma membrane integrity, acrosomal exocytosis, protein tyrosine phosphorylation, and motility (total motility and motion kinetics). The percentage of motile spermatozoa was determined after DGC (time 0) and at 30 and 180 min of incubation. Plasma membrane integrity, acrosomal exocytosis, and protein tyrosine phosphorylation were analyzed at time 0 and after 180 min of incubation.

After 30 min of incubation, HTF and HTF + PEN induced higher capacitation levels than WHI. Therefore, in the second experiment, spermatozoa were selected by thermotaxis after 30 min of incubation employing an in vitro system previously used in mouse and human sperm [22]. This selection was conducted for 60 min using a gradient from 35 to 38 °C (see the section below for details). Next, we determined the percentage of migration by thermotaxis. As migration by thermotaxis was only achieved using HTF + PEN, we analyzed in these samples the PTP of the migrating spermatozoa, non-migrating spermatozoa (those that did not migrate in the in vitro system), and unselected spermatozoa (aliquot incubated in parallel for 90 min at 37 °C in 5% CO_2_). In addition, DNA fragmentation was examined in the migrating and unselected spermatozoa.

### 2.3. Semen Collection and Cryopreservation

Semen was collected from six fertile purebred Lusitano stallions aged 3 to 13 years housed at the Centro de Selección y Reproducción Animal (CENSYRA) using an artificial vagina (Hannover model, Minitüb, Landshut, Germany). All experimental procedures were performed according to institutional and European regulations. A nylon in-line filter (Animal Reproduction Systems, Chino, CA, USA) was used to eliminate the gel fraction. The sperm-rich fraction was diluted 1:2 (*v:v*) in INRA96 medium (IMV, L’Aigle, France) and subsequently processed for cryopreservation. Diluted ejaculates were centrifuged for 10 min at 900× *g* and the supernatant discarded. The sperm pellet was re-suspended in an egg yolk-based freezing extender (Gent, Minitube Ibérica, Tarragona, Spain) to obtain a final concentration of 200 × 10^6^ sperm/mL, loaded into straws (0.5 mL) and sealed using sealing balls. Subsequently, the straws were equilibrated for 20 min at 4 °C and frozen by exposure to liquid nitrogen vapor at 4 cm above the liquid nitrogen level for 20 min. At the end of the cryopreservation process, the straws were submerged into liquid nitrogen at −196 °C where they were stored until analysis.

### 2.4. Sperm Sample Preparation and Incubation

Cryopreserved samples were thawed at 37 °C for 45 s in a water bath and processed by DGC. The contents of two straws were recovered into a microtube and transferred to a 15 mL centrifuge tube on top of 500 µL of equipure ^TM^ (Nidacon, Mölndal, Sweden) and centrifuged for 20 min at 400× *g*. Next, the supernatant was discarded and each pellet was resuspended in one of the following media: (i) WHI (100 mM NaCl, 4.7 mM KCl, 4.8 mM L-lactic acid hemicalcium salt, 1.2 mM MgCl_2_ × 6H_2_O, 5.5 mM glucose, 22 mM HEPES, and 1.0 mM pyruvic acid), (ii) HTF (2.04 mM CaCl_2_ × 2H_2_O, 101.6 mM NaCl, 4.69 mM KCl, 0.37 mM KH_2_PO_4_, 0.2 mM MgSO_4_ × 7H_2_O, 21.4 mM sodium lactate, 0.33 mM sodium pyruvate, and 2.78 mM glucose), or (iii) HTF supplemented with 750 µM of penicillamine (HTF + PEN). All media were supplemented with 25 mM NaHCO_3_, 4 mg/mL of bovine serum albumin (BSA), 100 U/mL penicillin, 50 µg/mL streptomycin SO_4_, and 0.001% (*w/v*) phenol red (pH = 7.4 and 280–300 mOsm/kg). Before their use, the media were preincubated overnight at 37 °C in a 5% CO_2_ humidified atmosphere. The sperm concentration was adjusted to 20 × 10^6^ spermatozoa/mL and samples were incubated for 3 h at 37 °C in a 5% CO_2_ humidifying atmosphere for capacitation. During the 3 h of incubation, pH was monitored and confirmed stable at 7.4.

### 2.5. Sperm Thermotaxis

Sperm thermotaxis was conducted as described elsewhere [22]. Briefly, our thermotaxis selection assay is based on recovering the spermatozoa who have migrated through a capillary between two drops of the same medium (in this experiment, HTF or HTF + PEN). For selection under thermotactic conditions, a 3 °C temperature gradient was set up between both drops from 35 to 38 °C. Between 5 and 6 × 10^6^ spermatozoa were loaded into the 35 °C drop and allowed to migrate for 1 h. After this time, migrated spermatozoa were recovered from the 38 °C drops and processed for tyrosine phosphorylation or DNA fragmentation analysis. As controls for random migration, two drops were placed in parallel to the thermotactic assay at the same temperature (35 to 35 °C and 38 to 38 °C, non-gradient controls). The percentage of net thermotaxis was calculated as follows: 100 × (number of spermatozoa migrating within the temperature gradient (35 to 38 °C) minus number of spermatozoa migrating within the temperature non-gradient (35 or 38 °C, selecting the temperature which resulted in higher random migration)/number of spermatozoa loaded].

### 2.6. Plasma Membrane Integrity

We employed propidium iodide (PI) to stain spermatozoa with damaged membranes. Sperm plasma membrane integrity was assessed using propidium iodide (PI) to stain spermatozoa with damaged membrane [25] and the fluorochrome Hoechst 33342 to stain the nuclei. Semen samples were diluted into PBS at a concentration of 2 × 10^6^ spermatozoa/mL, then PI and Hoechst 33342 were added to a final concentration of 10 and 15 µM, respectively. After 5 min, the stained samples were analyzed by flow cytometry in a FASCanto II flow cytometer (BD Biosciences, San Jose, CA, USA). Spermatozoa were gathered in the forward scatter and side scatter (FSC/SSC) dot plot to exclude debris and confirmed with the violet laser (405 nm) and the blue filter (450/50 nm) to detect nuclear staining with Hoechst 33342. A total of 1 × 10^4^ spermatozoa were acquired per determination. For PI, the blue laser (488 nm) and the orange filter (585/42 nm) were used. Acquired data were analyzed using FlowJo software (Becton–Dickinson, Franklin Lakes, NJ, USA) to determine the percentage of PI-stained spermatozoa per each sample.

### 2.7. Acrosomal Exocytosis

The method employed was based on acrosome staining using *Arachis hypogaea* (peanut) lectin conjugated with fluorescein isothiocyanate (PNA–FITC) following a standard protocol described previously [26], with minor modifications. Briefly, the spermatozoa were washed twice in fhosphate-buffered saline (PBS) by centrifugation (1 min at 500× *g*) and subsequently smeared on a microscope glass slide and air-dried on a heat plate at 37 °C. Next, the slides were immersed in absolute methanol for 30 s, air-dried, and rinsed in PBS twice for 5 min before incubation with PNA–FITC and Hoechst 33342 (15 µg/mL and 0.0065 mg/mL respectively, in H_2_O) in a wet-mount box/humidified box for 30 min at room temperature. Finally, the slides were washed with distilled water for 15 min and mounted with Fluoromount ^TM^ aqueous mounting medium. Slides were examined in a fluorescence microscope (Nikon Eclipse i50, Nikon, Tokyo, Japan) and numbers of acrosome-reacted and non-acrosome-reacted spermatozoa were counted by randomly moving across different fields of the slide (counting 200 cells per slide, 2 slides per sample).

### 2.8. Motility and Kinetics

Ten microliters of sperm suspension was placed in a Mackler chamber on the stage heated to 37 °C of a Nikon Eclipse E400 (Nikon, Tokyo, Japan) fitted with a digital camera, Basler acA1300-200uc (Basler AG, Ahrensburg, Germany). Three to five movies of 1.5 s were recorded at 60 frames/s using the software Pylon Viewer provided by Basler, capturing at least 100 moving spermatozoa. The motility and sperm kinetics were analyzed using the free software ImageJ 1.x [27] with the plugin CASA_bmg following instructions for analyzing stallion spermatozoa [28]. The parameters analyzed were as described by Mortimer et al. [29]: straight-line velocity (VSL; µm/s), curvilinear velocity (VCL; µm/s), average path velocity (VAP; µm/s), linearity (LIN) (defined as (VSL/VCL) × 100), straightness (STR) (defined as (VSL/ VAP) × 100), wobble (WOB) (defined as (VAP/VCL) × 100), amplitude of lateral head (ALH) displacement (µm), and beat-cross frequency (BCF; Hz). Also, we examined the percentage of spermatozoa showing more signs of hyperactivation (HYP) by determining out of all the analyzed spermatozoa (9340) the lower VCL and ALH values of the 10% of spermatozoa with the highest VCL and ALH. These values were: VCL = 150 µm/s and ALH = 5.5 µm. Thus, we defined spermatozoa showing hyperactive-like motility as those showing VCL > 150 µm/s and ALH > 5.5 µm (following the definition used in Su et al. [30]).

### 2.9. DNA Fragmentation

DNA fragmentation was analyzed employing the neutral version of the single cell gel electrophoresis assay (SCGE or Comet assay), as described previously [22]. Briefly, the samples were pelleted by centrifugation (600× *g*) and diluted to a maximum of 20 × 10^4^ spermatozoa/mL in 0.5% low melting point agarose in PBS. Because of the low numbers obtained in the thermotaxis assay, the samples of migrated spermatozoa were used entirely. Immediately after dilution, 85 µL were placed on a slide previously coated with 1% agarose and covered with a 22 × 22 mm coverslip. The slides were then left in a wet-mount box/humidified box at 4 °C for 1 h for agarose polymerization. After removing the coverslips, slides were incubated at 37 °C for 1 h in lysis solution (2 M NaCl, 55 mM EDTA-Na_2_, 8 mM Tris, 4% Triton X-100, 0.1% sodium dodecyl sulfate (SDS), 1 mM ditiotreitol (DTT), and 0.5 mg/mL of proteinase K, pH 8). Next, the slides were washed twice in neutral electrophoresis solution (90 mM Tris, 90 mM boric acid, and 2 mM EDTA, pH 8.5) and subjected to electrophoresis (25 V for 10 min). The slides were then washed with distilled water, fixed in methanol for 3 min, air-dried, and stored upon microscope examination. The samples were stained with 50 µL of 0.02 mg/mL ethidium bromide, covered with a 22 × 22 mm coverslip, and immediately observed in a fluorescence microscope Nikon Optiphot-2 (Nikon, Tokyo, Japan). Comets were digitalized with a Nikon 5100 digital camera (Nikon, Tokyo, Japan) coupled to the microscope. At least 150 comets were analyzed using the free software Casplab 1.2.3beta2 (CaspLab.com) [31].

### 2.10. Protein Tyrosine Phosphorylation

Protein tyrosine phosphorylation was analyzed by immunofluorescence. Spermatozoa were diluted in 500 µL of PBS to a concentration of 4 × 10^6^ spermatozoa/mL. Due to the low numbers obtained in the thermotaxis assay, the samples of migrated spermatozoa were used entirely and undiluted. Samples were centrifuged (600× *g* for 5 min) and the resultant pellet was fixed in 2% paraformaldehyde in PBS for 10 min and stored at –20 °C for, at most, one week, until continuing with immunodetection. After defrosting at room temperature, the fixed samples were washed 3 times in PBS by centrifugation (600× *g* for 5 min) and the pellet was resuspended in 50 µL of PBS. Two drops of 25 µL were each smeared on a glass microscope slide and left to dry. Subsequently, slides were washed three times with PBS and 100 µL of PBS with 0.2% of Triton-X were placed on each slide and covered with a 20 × 60 mm coverslip, placed in a wet box, and incubated at 37 °C for 15 min. Then, slides were washed once in PBS and incubated in a wet box with 100 µL of PBS and 1% BSA (again using a coverslip) for 1 h at 37 °C. Subsequently, slides were drained, and 100 µL of the primary antibody (phosphor-tyrosine monoclonal antibody (pY20), reference 14-5001-82, ThermoFisher Scientific, Waltham, MA, USA) diluted 1:100 in PBS was added to each slide, covered with a coverslip, and incubated overnight at 4 °C. On the next day, slides were washed three times in PBS and the secondary antibody (goat anti-mouse IgG (H + L) highly cross-adsorbed secondary antibody, Alexa Fluor 488, reference A-11029, ThermoFisher Scientific, Waltham, MA, USA) was added (100 µL of a 1:100 dilution in PBS and covered with a coverslip) and incubated for 1 h in a wet box at 37 °C. Slides were then washed three times in PBS and nuclei-counterstained with 100 µL of 15 µM Hoechst 33342 by incubating for 5 min in a wet box. After an additional wash in PBS, the slides were mounted with Fluoromount ^TM^ aqueous mounting medium and examined in a fluorescence microscope (Nikon Eclipse i50, Nikon, Tokyo, Japan). Numbers of tyrosine-phosphorylated spermatozoa were counted by randomly moving across different fields of the slide (counting 200 cells per slide, 2 slides per sample).

### 2.11. Statistical Analysis

Statistical analysis was carried out using the software package GraphPad Prism 8.0.2 for Windows (GraphPad Software, San Diego, CA, USA). Results are expressed as means ± standard error of the mean (SEM). Means were compared and analyzed using a one-tailed paired-sample Student’s *t*-test or repeated measures one-way analysis of variance (ANOVA), followed by Tukey’s post hoc test. Significance was set at *p* < 0.05.

## 3. Results

**Figure 1:**
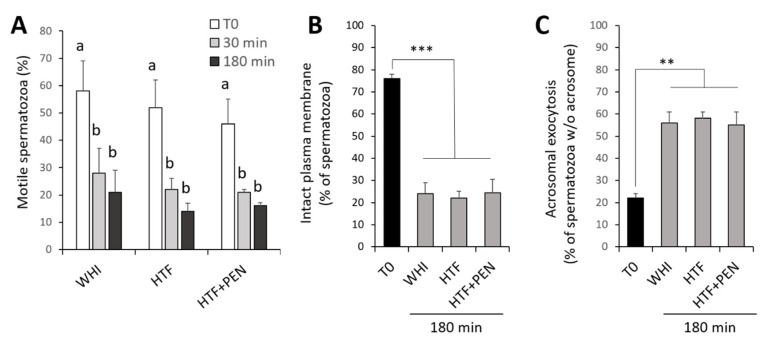
Sperm integrity over a 180 min period of capacitation. (**A**) Percentage of motile spermatozoa. (**B**) Percentage of spermatozoa with an intact plasma membrane as confirmed by propidium iodide staining. (**C**) Percentage of spermatozoa showing acrosomal exocytosis as determined by *Arachis hypogaea* (peanut) lectin conjugated with fluorescein isothiocyanate (PNA-FITC) staining. Spermatozoa after density gradient centrifugation (T0) and incubation for 30 or 180 min under capacitating conditions in three media: Whitten’s medium (WHI), synthetic human tubal fluid (HTF), and HTF supplemented with 750 µM of penicillamine (HTF + PEN). *** *p* < 0.0001, ** *p* < 0.0019; ^a,b^ different letters indicate significant differences (*p* < 0.05), (*n* = 6, 12 determinations).

We employed frozen/thawed spermatozoa from 6 stallions that were prepared by density gradient centrifugation and incubated for 180 min in WHI, HTF, or HTF + PEN. To assess sample integrity, we determined percentage motility, plasma membrane integrity, and the occurrence of acrosomal exocytosis before and during incubation. The percentage of motile spermatozoa was similar between the three studied media at every time point during incubation, with a significant decrease detected in the initial 30 min (Figure 1A) (*p* < 0.002). From 30 to 180 min of incubation, motility slightly diminished, though not significantly (*p* > 0.38). Incubation with the three media for 180 min also provoked a significant reduction in the percentage of spermatozoa showing an intact plasma membrane (Figure 1B) and a significant increase in spermatozoa undergoing acrosomal exocytosis (Figure 1C) (*p* < 0.0001 and *p* < 0.0019, respectively).

### 3.1. Effects of the Incubation Medium on Sperm Kinetics and Protein Tyrosine Phosphorylation

**Figure 2:**
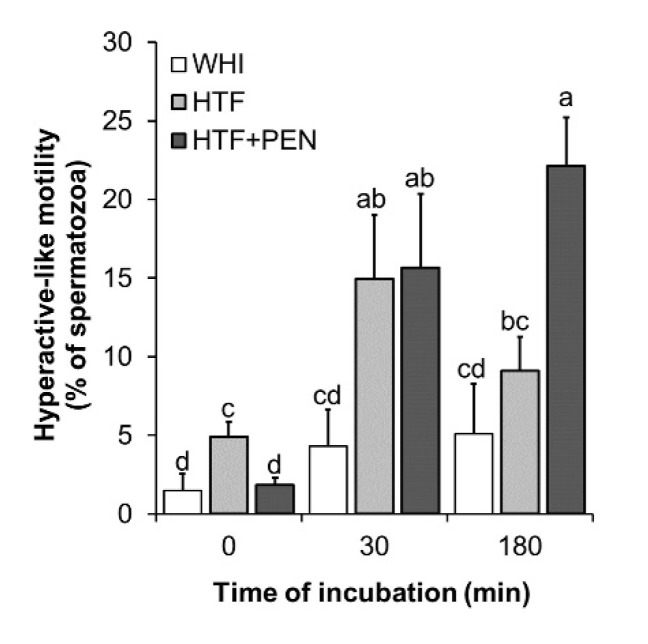
Percentage of spermatozoa showing hyperactive-like motility. Kinetics were examined at the time points 0, 30, and 180 min of incubation in Whitten’s medium (WHI), synthetic human tubal fluid (HTF), or HTF supplemented with 750 µM of penicillamine (HTF + PEN). Spermatozoa showing hyperactive-like motility were defined as those showing VCL > 150 and ALH > 5.5. ^a, b, c, d^ Different letters indicate significant differences (*p* < 0.05, *n* = 6, 12 determinations).

To determine which of the media, WHI or HTF, could potentially induce more spermatozoa to acquire the capacitation state, we conducted a comparative analysis of sperm kinetics and protein tyrosine phosphorylation over the 180 min of incubation. At the onset of incubation, VCL and ALH of swimming spermatozoa were higher in the HTF medium than WHI (*p* = 0.045 and *p* = 0.04, respectively) (Table 1). Thus, in HTF, we detected a higher fraction of spermatozoa showing a motility classified as indicating hyperactivation (relatively high VCL (>150 µm/s) and ALH (>5.5 µm), hereafter referred to as hyperactive-like motility) (*p* = 0.02) (Figure 2). Moreover, sperm kinetics in WHI did not significantly vary during the 180 min of incubation, while in HTF, sperm gradually acquired a less progressive motility type (LIN and STR reduced after 30 min (*p* = 0.04 and *p* = 0.01, respectively), as well as a decrease in WOB and beat cross frequency detected after 180 min (*p* = 0.04 and *p* = 0.014). These motion changes produced during incubation in HTF were recorded as a significant increase in the percentage of spermatozoa showing hyperactive-like motility after 30 min of incubation (*p* = 0.045) (Figure 2). However, after 180 min, hyperactive-like motility returned to the levels observed at the start of incubation.

**Figure 3:**
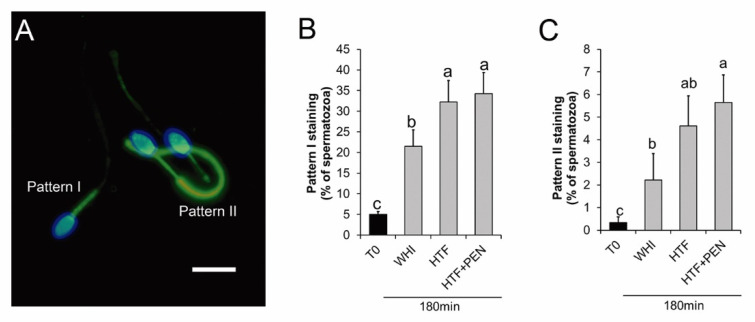
Protein tyrosine phosphorylation in stallion spermatozoa after 180 min of incubation for capacitation. (**A**) Micrograph of fluorescence microscopy of stallion spermatozoa after 180 min of capacitation and immune-labelled for protein tyrosine phosphorylation (PTP) (green). Nuclei were labelled with Hoechst 33342 (blue), bar = 10 µm. Pattern I: spermatozoa showing PTP at the midpiece. Pattern II: Spermatozoa showing PTP along the whole flagellum. (**B**,**C**) Percentage of spermatozoa showing pattern I (B) and pattern II (**C**) staining at time 0 and after 180 min of incubation in Whitten’s medium (WHI), synthetic human tubal fluid (HTF), or HTF supplemented with 750 µM of penicillamine (HTF + PEN). ^a, b, c^ Different letters indicate significant differences (*p* < 0.05, *n* = 6, 12 determinations).

Immunofluorescence PTP analyses revealed two staining patterns of the flagella: (i) staining showing PTP only in the midpiece (pattern I) and (ii) staining showing PTP along the whole flagellum (pattern II) (Figure 3A). The percentage of spermatozoa showing either staining pattern increased during incubation in the two media (Figure 3B,C). This increase was significantly higher for pattern I when the incubation medium was HTF rather than WHI (*p* = 0.027) (Figure 3B). No significant differences in pattern II emerged between HTF and WHI (*p* = 0.1) (Figure 3C).

### 3.2. Effects of HTF Supplementation with Penicillamine on Sperm Kinetics and Protein Tyrosine Phosphorylation

To examine the effect of PEN on capacitated spermatozoa, it was added to HTF at a final concentration of 750 µM [20] and sperm kinetics and PTP were analyzed over the 180 min of incubation. At the start of incubation, we detected no significant differences in kinetics for HTF versus HTF + PEN (Table 1). However, at this early stage, the percentage of spermatozoa showing hyperactive-like motility was higher for HTF (*p* = 0.01). Interestingly, after 30 min of incubation, kinetics and hyperactive-like motility were similar in both media, but after 180 min, the spermatozoa incubated in HTF + PEN swam with significantly higher VCL (*p* = 0.03), VAP (*p* = 0.033), and ALH (*p* = 0.04), and lower STR (*p* = 0.02). Accordingly, the percentage of spermatozoa showing hyperactive-like motility after 180 min of incubation was higher in HTF + PEN (*p* = 0.004) (Figure 2). Our immunofluorescence analyses, nevertheless, revealed no differences in PTP for both staining patterns (Figure 3B,C). Compared to incubation in HTF alone, supplementation with PEN gave rise to a significantly higher percentage of spermatozoa showing PTP staining pattern II compared to WHI (*p* = 0.04) which, in turn, could indicate a slightly higher incidence of PTP related to incubation in HTF + PEN compared to HTF alone.

### 3.3. Sperm Thermotaxis

**Figure 4:**
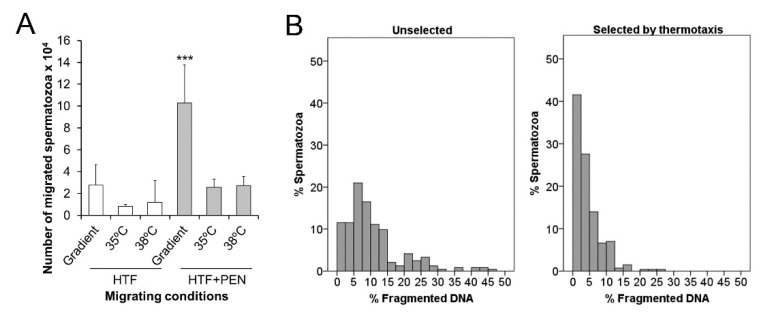
Sperm selection by thermotaxis. (**A**) The number of spermatozoa in vitro migrating from 35 to 38 °C or across a constant temperature (35 or 38 °C) for 60 min after 30 min of incubation in synthetic human tubal fluid (HTF) or HTF supplemented with 750 µM of penicillamine (HTF + PEN). The initial number of spermatozoa loaded in the thermotaxis system was between 5 and 6 × 10^6^ per separation. (**B**) Histograms show the distributions of % fragmented DNA in individual spermatozoa unselected or selected by thermotaxis after incubation for 30 min in HTF + PEN. *** *p* = 0.009, *n* = 6.

As thermotaxis is a capacitation-dependent process that requires spermatozoa to maintain their swimming capacity to migrate within a temperature gradient, we employed our in vitro thermotaxis assay to analyze the effect of PEN in prolonging the migration ability of capacitated spermatozoa. When incubated in HTF + PEN and not HTF alone, the number of migrated spermatozoa within the temperature gradient was significantly higher compared to those migrating in the absence of a gradient (constant temperature of 35 or 38 °C) (*p* < 0.002) (Figure 4A). These results confirm the occurrence of thermotaxis, the percentage of net thermotaxis being 1.1% ± 0.5% for HTF + PEN (percentage of spermatozoa migrated in vitro by thermotaxis referred to the loaded spermatozoa) and confirmed the protective effect of PEN supplementation. Further, as thermotaxis allows for the selection of a sperm subpopulation of high genetic integrity in humans and mice [22], we analyzed DNA fragmentation of the selected spermatozoa. Our results indicate significantly lower DNA fragmentation in selected spermatozoa compared to the unselected sample (aliquot incubated in parallel at 37 °C) (4.4% ± 0.4% and 11% ± 2% respectively, *p* = 0.009). These selected spermatozoa fractions also showed enrichment in populations with low DNA fragmentation (Figure 4B). Thus, the percentage of spermatozoa showing 0–5% DNA fragmentation (high DNA integrity) was significantly higher in the selected fraction (69% ± 4% vs 23% ± 4% respectively, *p* = 0.0002).

**Figure 5:**
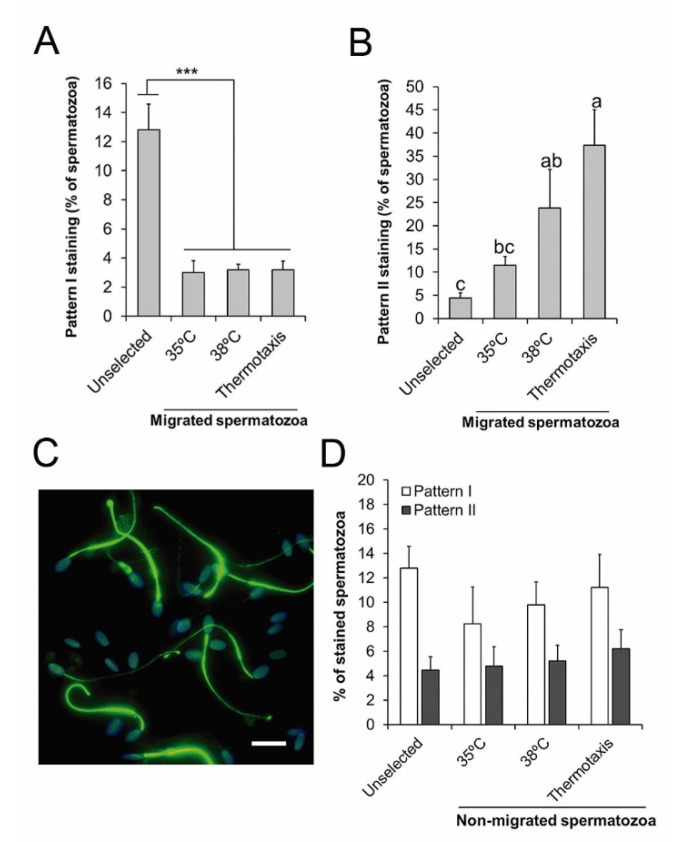
Protein tyrosine phosphorylation in the thermotaxis assay. (**A**,**B**) Percentage of spermatozoa showing staining patterns I or II for tyrosine phosphorylation in the unselected sample, in the spermatozoa migrating across constant temperatures (35 or 38 °C), or across a temperature gradient (from 35 to 38 °C, thermotaxis). (**C**) Representative micrograph of immunofluorescence for tyrosine phosphorylation (green) in stallion spermatozoa migrating by thermotaxis. Nuclei were labelled with Hoechst 33342 (blue). Bar = 10 µm. (**D**) Percentage of spermatozoa showing staining patterns I and II for protein tyrosine phosphorylation in the unselected sample or in those spermatozoa that did not migrate during the thermotaxis assay in the conditions described above. *** *p* = 0.001, ^a, b, c^ different letters indicate significant differences (*p* < 0.05, *n* = 6).

To examine the relationship between PTP and the ability of the spermatozoa to migrate by thermotaxis, we also conducted PTP immunofluorescence analyses on selected and unselected spermatozoa. Our results revealed lower percentages of spermatozoa showing the PTP staining pattern I in the migrated spermatozoa (in all the thermotaxis and both non-gradient controls) compared to the unselected sample (*p* < 0.0002) (Figure 5A). In contrast, the percentage of spermatozoa showing PTP staining pattern II was significantly higher in the spermatozoa migrating in the non-gradient control at 38 °C and in those spermatozoa selected by thermotaxis when compared to the unselected sample (*p* < 0.02) (Figure 5B,C). In the non-migrating spermatozoa (those remaining in the drop where they were first loaded in the thermotaxis system), percentages of PTP staining patterns I and II were similar for all the conditions analyzed and in the unselected sample (Figure 5D).

## 4. Discussion

Several hypotheses have been put forward to explain the unsuccessful in vitro capacitation of stallion spermatozoa [1]. The results of our study along with the literature findings detailed below suggest that the media employed should be rethought by optimizing concentrations of energy sources and adding supplements to modulate and prolong the lifespan of spermatozoa once capacitated. In our study, the HTF medium induced the greater occurrence of capacitation-related events during incubation when compared to WHI, presumably because of its higher lactate content. We also observed that supplementation with PEN prolonged the duration of hyperactive-like motility and this allowed the sperm migration by thermotaxis, suggesting a pro-survival effect on the capacitated sperm population. Interestingly, we also found that spermatozoa selected by thermotaxis showed relatively good DNA integrity, corroborating our previous results in the mouse and human [22] and opening the possibility of employing this method to improve ARTs. Another significant result that we also discuss here was the lack of a relationship found between PTP in the whole flagellum and sperm migration by thermotaxis. This might indicate the existence of a physiological PTP-independent hyperactivation response.

### 4.1. Capacitation-Related Events During Incubation in HTF

Incubation in HTF led to a time-dependent effect on sperm kinetics whereby hyperactive-type motility was acquired by a fraction of the spermatozoa. This effect was not observed when spermatozoa were incubated in WHI, which is a medium commonly used for stallion sperm capacitation. Further, although both media significantly increased PTP levels after 180 min of incubation, higher levels were attained with HTF. This difference was not detected by Arroyo-salvo et al. [8], who conducted a similar comparative study. In contrast, they found no time-dependent changes in sperm kinetics compatible with hyperactivation over 120 min, and PTP induction levels were similar to both media after 120 and 240 min of incubation. However, Arroyo-salvo et al. [8] employed a fresh sample washed by centrifugation, while we used frozen/thawed samples washed by DGC, which could explain the differences between both studies. Cryopreservation provokes significant changes in the sperm plasma membrane, increasing membrane peroxidation and permeability that could trigger capacitation-related events [32,33]. Further, even if capacitation is not immediately triggered, freezing/thawing may leave the spermatozoa in a poised status, making them more susceptible to capacitation than fresh sperm, as confirmed elsewhere [34]. We also detected the significant occurrence of spontaneous acrosomal exocytosis after 180 min of incubation, not detected by others employing fresh stallion semen [3,8]. This also supports the higher susceptibility of frozen/thawed spermatozoa to the destabilizing changes occurring during capacitation, as has been also shown for bull spermatozoa [35].

Differences in composition between WHI and HTF could explain the observed differences in both sperm kinetics and PTP. Both media differ in the amount of glucose and pyruvate they contain, and these are ~2 and 3 times less concentrated in HTF, respectively. However, HTF contains ~4.5 times more lactate than WHI (21.4 and 4.8 mM, respectively), which can be directly transformed to pyruvate in the sperm mitochondria by the Krebs cycle [36]. In effect, lactate and pyruvate are the main sources of energy utilized by stallion spermatozoa, and glucose may even reduce mitochondrial function [37]. Thus, the higher concentration of an energy source that can be rapidly and effectively utilized by the mitochondria could increase their activity [37], enhancing ROS production which will subsequently trigger PTP and its associated hyperactive-like motility [9]. Hence, a greater mitochondrial activity could explain the higher VCL and hyperactive-like motility observed from the onset of incubation and the higher PTP levels reported here after 180 min when using HTF rather than WHI. This could also explain the effect observed in enhancing kinetics when incubating stallion spermatozoa with follicular fluid from pre-ovulatory follicles [38]. As examined in buffalo, bull, sheep, rat, and mouse, this fluid also contains higher concentrations of lactate than glucose + pyruvate, ranging from ~7 to 27 mM depending on the species [39,40,41]. In humans, similar levels of glucose and lactate are reported, of around 3 mM [42]. Recently González-Fernández et al. [43] reported that in the mare’s pre- and post-ovulatory oviductal fluids, concentrations of lactate were 54.66 ± 10.7 and 69.25 ± 7.3 mM, while concentrations of glucose were 0.18 ± 0.04 and 0.57 ± 0.2 mM, respectively. It is also important to point out that lactate is the most abundant source of energy within the oviduct [43,44] where capacitation, and thus hyperactivation, is triggered in vivo [45].

### 4.2. Effect of Penicillamine on Capacitation-Related Events During Incubation with HTF

Under our capacitating conditions, PEN was not able to rescue the time-dependent loss of motility and plasma membrane integrity. This contrasts with the results reported by Aitken et al. [20], where PEN prolonged the motility of fresh sperm incubated in BWW medium without BSA. The pro-survival effect of PEN on spermatozoa has been attributed to its ability to inactivate and slow down the production of cytotoxic aldehydes by lipid peroxidation provoked by ROS produced by the mitochondria [20]. As capacitation enhances mitochondrial function, ROS production is increased and modulates intracellular signaling for sperm capacitation, stimulating adenylyl cyclase and inhibiting tyrosine phosphatases, causing a downstream increase in PTP [9,46]. However, when oxidative stress exceeds a certain limit, the spermatozoa undergo an apoptotic-like process [18]. Thus, in our experiment, the sperm fraction that abruptly lost motility within the first 30 min of incubation and lost membrane integrity after 180 min of incubation, could have exceeded ROS production, overwhelming the protective effect of PEN. Another option is that a different deleterious process associated with sperm capacitation may have compromised sperm lifespan in a fraction of our samples. The significant changes observed in the architecture of the plasma membrane produced during capacitation, such as cholesterol removal, glycoprotein redistribution, and loss of phospholipid asymmetry [47], affects the lifespan of the spermatozoa, making them more vulnerable to damage. Thus, the plasma membrane of capacitated spermatozoa becomes more permeable to vital stains such as propidium iodide [48] and/or, as occurs with the acrosomal membrane, becomes more prone to destabilization [49]. This was likely more pronounced in our samples as we employed frozen/thawed spermatozoa, which are known to be more sensitive to the destabilizing conditions of capacitation, as the abundance of spermatozoa sustaining sublethal damage could be high. In agreement, Pommer et al. [34] showed that, in contrast to fresh sperm, frozen/thawed stallion spermatozoa incubated under capacitating conditions lost motility and membrane integrity within an hour of incubation.

We found that, unlike the case of HTF alone, supplementation with PEN led to a sustained fraction of motile spermatozoa (between 16% ± 5% and 22% ± 3%) with relatively high VCL and ALH, indicating hyperactive-like motility from 30 to 180 min of incubation. Using this medium, after 180 min, PTP along the whole flagellum reached 6% ± 1% of the total spermatozoa, from close to 0 at the onset of incubation. Assuming that only motile spermatozoa (20% ± 2% after 30 min of incubation with HTF + PEN) will acquire the capacity for PTP during incubation, then the percentage of PTP on the whole flagellum within the motile population may represent some 30%. Thus, we hypothesize that the spermatozoa that showed hyperactive-like motility could be those undergoing PTP in the whole flagellum [10,11], as each indicator was present in similar percentages of spermatozoa. No differences in PTP were observed when we compared the use of HTF + PEN to HTF alone, indicating that PEN did not induce more spermatozoa to enter a capacitated-like state, but protected those that did and also lengthened the duration of this acquired hyperactive-like motility. As commented above, physiological ROS are needed to induce hyperactivation in human sperm [50,51], but as a consequence, oxidative stress generates cytotoxic aldehydes, damaging the cell [20]. The first structure injured by this oxidative stress is the mitochondrial membrane, thus motility is the first function affected [52,53]. Accordingly, the protective effect of PEN in this setting has been shown to enhance the velocity of the motile sperm fraction in horse, rat, and human [20]. In our experiment, we also found that after 180 min of incubation in HTF + PEN, spermatozoa showed significantly higher VCL than in HTF alone. Thus, our results suggest that PEN was able to maintain the observed hyperactive-like motility, possibly prolonging the lifespan of the capacitated spermatozoa fraction. Our theory is in line with the results reported by Pavlok [21], in which PEN prolonged the fertilizing ability of frozen/thawed bovine spermatozoa.

### 4.3. Penicillamine Enables Sperm Selection by Thermotaxis

To carry out thermotaxis, spermatozoa must be motile and capacitated so that they can move across the temperature gradient [23,24]. Thus, for the thermotaxis experiments, spermatozoa were capacitated for 30 min in HTF or HTF + PEN, as at this time point, we had observed hyperactive-like motility with both media. However, only when incubated in HTF + PEN were spermatozoa able to migrate by thermotaxis. This observation supports the protective effect of PEN on the fraction of capacitated spermatozoa. Thus, in addition to maintaining the specific sperm kinetics needed for migration, PEN could be protecting intracellular signaling involved in the thermotactic response itself. This signaling is mediated by the phosphodiesterase and phospholipase C pathways whose thermosensors are thought to be opsins [54,55] as well as transient receptor potential cation channel subfamily V member 1 (TRPV1) [56].

In the HTF + PEN medium, thermotaxis selection yielded a net thermotaxis of 1.1% ± 0.5%, similar to the response reported in humans and mice using the same protocol [22] or employing other devices [54]. Our DNA damage assessment revealed that the fraction migrating by thermotaxis was significantly enriched in spermatozoa bearing high DNA integrity, as reported for human and mouse sperm [22]. As suggested for these two species and now also for horses, thermotaxis might be a bi-functional mechanism for the navigation and selection of high-quality capacitated spermatozoa in mammals.

Both the fraction of spermatozoa selected by thermotaxis and the fraction of spermatozoa showing random movement at a constant temperature of 38 °C showed similar strong enrichment in spermatozoa with PTP along the whole flagellum (37% ± 8% and 24% ± 8%, respectively). These percentages are similar to the percentage of PTP in the whole flagellum estimated above for the motile fraction (~30%), assuming that only motile spermatozoa can trigger PTP in the whole flagellum. This suggests that thermotaxis selects a fraction within the motile spermatozoa independently of the PTP status of the flagellum and argues against the involvement of PTP-dependent hyperactivation in the behavioral thermotaxis response of spermatozoa. Further, the lower levels of PTP detected in the sample of spermatozoa migrating in the non-gradient control at 35 °C indicates a direct PTP-inducing effect of temperature within the motile and migrating sperm fraction. This direct relationship between absolute temperature and PTP is a well-known phenomenon [57] and could indicate that PTP in the thermotactic fraction occurs during spermatozoa migration or once they have migrated. Boryshpolets et al. [57] reported that changes in the direction of swimming during the thermotactic response of human spermatozoa occur as turns that may be subtle or generated by episodes of hyperactivation. Thus, the model proposed by Boryshpolets et al. [58] for the thermotactic behavior response implies that hyperactive-like motility is transient and more frequent at lower temperatures. This contrasts with the longstanding nature of hyperactivation related to flagellum PTP and explains why in our experiment there was no significant PTP enrichment in the spermatozoa migrated by thermotaxis compared to those moving across the non-gradient control at 38 °C. We, therefore, propose the hyperactive-like motility involved in thermotaxis is PTP-independent and possibly directly linked to opsin and TRPV1 signaling for a rapid transient response. As support for this theory of PTP-independent hyperactive motility, procaine and caffeine have been shown to induce hyperactive-like motility in stallion and ram spermatozoa independently of PTP, respectively [59,60]. Further work is needed to elucidate the full transduction signaling pathway coupled to the sperm temperature sensing machinery along with the behavior changes involving the transient acquisition of hyperactive-like motility.

## 5. Conclusions

In this study, we observed a protective effect of penicillamine used for the in vitro capacitation of stallion spermatozoa in prolonging the duration of hyperactive-like motility of a fraction of the sperm sample and in allowing sperm migration by thermotaxis, a process that is capacitation-dependent. In addition, we report here that thermotaxis selects a sperm fraction enriched in PTP also showing high DNA integrity, thus supporting its potential use for sperm preparation before assisted reproductive techniques in the horse. The results reported here also point to a relevant role of lactate in the capacitation of stallion spermatozoa and also identify no relationship between protein tyrosine phosphorylation in the sperm flagellum and migration by thermotaxis.

## Figures and Tables

**Table 1:** Kinetics of stallion spermatozoa measured at time 0 (T0), and after 30 and 180 min of incubation at 37°C in a 5% CO_2_ atmosphere in Whitten’s medium (WHI), synthetic human tubal fluid (HTF), and HTF supplemented with 750 µM of penicillamine (HTF + PEN).

Kinetics	T0			30 min			180 min		
	WHI	HTF	HTF + PEN	WHI	HTF	HTF + PEN	WHI	HTF	HTF + PEN
VCL (µm/s)	80 ± 6	96 ± 5 *	97 ± 2 ^a^ *	87 ± 7	109 ± 7 *	114 ± 5 ^b^ *	81 ± 7	107 ± 4 *	122 ± 3 ^b^ **
VAP (µm/s)	36 ± 1	40 ± 2	40 ± 0.8 ^a^	37 ± 1	44 ± 3	45 ± 2 ^b^	35 ± 2	41 ± 2	47 ± 1 ^b^ *
VSL (µm/s)	28 ± 2	30 ± 1	29 ± 1	27 ± 1	26 ± 3	27 ± 1	24 ± 2	25 ± 2	25 ± 2
LIN (%)	37 ± 4	33 ± 2 ^a^	31 ± 2 ^a^	34 ± 2	25 ± 3 ^b^	25 ± 1 ^b^	30 ± 1	24 ± 2 ^b^ *	21 ± 1 ^c^ *
STR (%)	78 ± 4	75 ± 3 ^a^	71 ± 3 ^a^	74 ± 2	59 ± 7 ^b^ *	61 ± 3 ^b^ *	64 ± 4	61 ± 4 ^b^	55 ± 3 ^b^ *
WOB (%)	46 ± 2	43 ± 1 ^a^	43 ± 1 ^a^	44 ± 3	41 ± 1 ^a^	40 ± 1 ^a b^	46 ± 2	39 ± 1 ^b^	39 ± 1 ^b^
Beat Cross (Hz)	24.2 ± 1.4	24 ± 0.5 ^a^	23.7 ± 0.5	25.3 ± 1	26 ± 0.5 ^a b^	24.2 ± 0.7	26.1 ± 0.9	27 ± 1 ^b^	24.7 ± 1.1
ALH (µm)	3 ± 0.2	3.5 ± 0.2 *	3.5 ± 0.1 ^a^ *	3.2 ± 0.2	3.9 ± 0.3 *	4.1 ± 0.2 ^b^ *	2.9 ± 0.2	3.8 ± 0.2 *	4.4 ± 0.1 ^b^ **

Curvilinear velocity (VCL), average path velocity (VAP), straight line velocity (VSL), linearity (LIN), straightness (STR), wobble (WOB), and amplitude of lateral head displacement (ALH). ^a, b^ Different letters indicate significant differences between time points for each medium. Asterisks indicate significant differences between media for each time point (*p* < 0.05, repeated measures one-way analysis of variance (ANOVA)).
